# Pediatric melioidosis in Sarawak, Malaysia: Epidemiological, clinical and microbiological characteristics

**DOI:** 10.1371/journal.pntd.0005650

**Published:** 2017-06-09

**Authors:** Anand Mohan, Yuwana Podin, Nickson Tai, Chae-Hee Chieng, Vanessa Rigas, Barbara Machunter, Mark Mayo, Desiree Wong, Su-Lin Chien, Lee-See Tan, Charles Goh, Reginal Bantin, Alexander Mijen, Wen-Yi Chua, King-Ching Hii, See-Chang Wong, Hie-Ung Ngian, Jin-Shyan Wong, Jamilah Hashim, Bart J. Currie, Mong-How Ooi

**Affiliations:** 1 Department of Pediatrics, Bintulu Hospital, Bintulu, Sarawak, Malaysia; 2 Institute of Health and Community Medicine, Universiti Malaysia Sarawak, Kota Samarahan, Sarawak, Malaysia; 3 Global and Tropical Health Division, Menzies School of Health Research, Charles Darwin University, Northern Territory, Australia; 4 Department of Pediatrics, Kapit Hospital, Kapit, Sarawak, Malaysia; 5 Department of Pediatrics, Sibu Hospital, Sibu, Sarawak, Malaysia; 6 Department of Pathology, Kapit Hospital, Kapit, Sarawak, Malaysia; 7 Department of Pathology, Bintulu Hospital, Bintulu, Sarawak, Malaysia; 8 Department of Pathology, Sibu Hospital, Sibu, Sarawak, Malaysia; 9 Kapit Divisional Health Department, Kapit, Sarawak, Malaysia; 10 Internal Medicine Unit, Borneo Medical Centre, Kuching, Sarawak, Malaysia; 11 Department of Medicine, Faculty of Medicine and Health Sciences, Universiti Malaysia Sarawak, Kota Samarahan, Sarawak, Malaysia; 12 Sarawak State Health Department, Kuching, Sarawak, Malaysia; 13 Department of Pediatrics, Sarawak General Hospital, Kuching, Sarawak, Malaysia; University of Tennessee, UNITED STATES

## Abstract

**Background:**

Melioidosis is a serious, and potentially fatal community-acquired infection endemic to northern Australia and Southeast Asia, including Sarawak, Malaysia. The disease, caused by the usually intrinsically aminoglycoside-resistant *Burkholderia pseudomallei*, most commonly affects adults with predisposing risk factors. There are limited data on pediatric melioidosis in Sarawak.

**Methods:**

A part prospective, part retrospective study of children aged <15 years with culture-confirmed melioidosis was conducted in the 3 major public hospitals in Central Sarawak between 2009 and 2014. We examined epidemiological, clinical and microbiological characteristics.

**Findings:**

Forty-two patients were recruited during the 6-year study period. The overall annual incidence was estimated to be 4.1 per 100,000 children <15 years, with marked variation between districts. No children had pre-existing medical conditions. Twenty-three (55%) had disseminated disease, 10 (43%) of whom died. The commonest site of infection was the lungs, which occurred in 21 (50%) children. Other important sites of infection included lymph nodes, spleen, joints and lacrimal glands. Seven (17%) children had bacteremia with no overt focus of infection. Delays in diagnosis and in melioidosis-appropriate antibiotic treatment were observed in nearly 90% of children. Of the clinical isolates tested, 35/36 (97%) were susceptible to gentamicin. Of these, all 11 isolates that were genotyped were of a single multi-locus sequence type, ST881, and possessed the putative *B*. *pseudomallei* virulence determinants *bimA*_Bp_, *fhaB3*, and the YLF gene cluster.

**Conclusions:**

Central Sarawak has a very high incidence of pediatric melioidosis, caused predominantly by gentamicin-susceptible *B*. *pseudomallei* strains. Children frequently presented with disseminated disease and had an alarmingly high death rate, despite the absence of any apparent predisposing risk factor.

## Author summary

Melioidosis is a serious, and often fatal community-acquired infection endemic to Southeast Asia and northern Australia. It is caused by the environmental saprophyte *Burkholderia pseudomallei*, a bacterium that is intrinsically resistant to many commonly used antibiotics. Its presence in Sarawak, Malaysian Borneo, has been documented, and recently, a novel gentamicin-susceptible strain discovered. However, there are limited data on the burden and clinical characteristics of melioidosis in Sarawak, both in adults and in children. In this study, we comprehensively investigated all pediatric melioidosis cases in Central Sarawak over a 6-year period. We found that this region has a very high incidence of pediatric melioidosis, and that children frequently presented with disseminated disease and had high fatality rates, despite the absence of any predisposing risk factor. We confirmed that these infections were caused predominantly by gentamicin-susceptible *B*. *pseudomallei* strains. We also highlighted other undescribed epidemiological, clinical and microbiological features, which may help in the overall understanding of *B*. *pseudomallei* infections. We emphasized the importance of improving the awareness and recognition of melioidosis in children, both in Sarawak and in other endemic regions.

## Introduction

Melioidosis is a serious tropical infectious disease endemic to Southeast Asia and northern Australia, caused by the gram-negative bacillus *Burkholderia pseudomallei* [1]. The bacterium, found in soil and surface water [2], is acquired through inoculation, inhalation and ingestion [3]. In endemic regions, melioidosis is a major cause of fatal community-acquired bacteremia and pneumonia in adults [4, 5], and case fatality rates of 50% continue to be reported [6].

Pediatric melioidosis is reported to be less common than adult disease, constituting between 5–15% of all melioidosis cases [7]. Children are less likely to develop disseminated disease, and deaths are believed to occur mainly in individuals with predisposing risk factors [8]. Epidemiologically separate scenarios are neonatal melioidosis and also transmission to infants from breast milk of mothers with melioidosis mastitis, both with high mortality [9, 10]. Although the majority of pediatric melioidosis data to date have originated from small, retrospective studies, marked differences in disease manifestations have been noted between the various melioidosis endemic regions. For instance, while parotid infection occurs in 25% of childhood infections in Thailand and neurological disease is extremely uncommon [11], the salivary gland is rarely involved in northern Australia but neurological disease occurs in as many as 38% of cases [12]. In Malaysia, both parotid and neurological involvement have been reported [13, 14]. Regional variations in acquisition route, host immune response and bacterial strain genetic factors may be contributing to these differences in disease manifestations [3].

Sarawak, in Malaysian Borneo, is endemic for melioidosis; although data have largely been limited to seroprevalence studies and case reports in adults [15, 16]. Of note, a recent study from Central Sarawak reported an unusually high isolation rate of gentamicin-susceptible *B*. *pseudomallei* [17], whereas the hallmark of *B*. *pseudomallei* has been intrinsic resistance to aminoglycosides [18].

The burden and the clinical characteristics of pediatric melioidosis in Sarawak have never been documented. In addition, the importance of the recently reported gentamicin-susceptible *B*. *pseudomallei*, and the molecular characteristics of *B*. *pseudomallei* in this region, remain unknown. To answer these questions, we conducted a part prospective, part retrospective study to determine the epidemiological, clinical and microbiological characteristics of pediatric melioidosis in Central Sarawak.

## Methods

### Study sites and population

The study was conducted at the 3 major public hospitals (Sibu, Bintulu and Kapit Hospital) that provide medical, surgical and intensive care services for adults and children living in Central Sarawak; an area of approximately 60,000 km^2^ with a total population of 592,000, including 173,000 aged <15 years.

We prospectively identified all children aged <15 years with culture-confirmed melioidosis presenting to the pediatric or intensive care ward from July 2010 to December 2014. Details on demography, underlying medical conditions, symptoms, physical findings, laboratory results, case management and outcome were collected using standardized data collection forms. Medical records of culture-confirmed melioidosis cases diagnosed between January 2009 and June 2010 were also retrieved and relevant data were recorded using the same data collection forms.

### Case definitions

Melioidosis was classified as either localized or disseminated. A localized disease was defined as a single, discrete, culture-positive focus of infection, in the absence of a positive blood culture and clinical/radiological evidence of dissemination to other sites. Disseminated disease was defined as the presence of infection in ≥2 discrete body sites and/or a positive blood culture. Infection sites were established based on physical and/or radiological findings. The primary infection site was determined based on the initial organ-specific symptom. Secondary sites were infection sites that developed subsequently or only became evident after hospital admission. Based on admission body weight, children were defined as having poor nutritional status if they were moderately or severely underweight. Children aged ≤10 years were categorized as moderately or severely underweight based on World Health Organization definitions [19], while children aged >10 years were considered at least moderately underweight if their body weight was below the 3rd percentile [20]. Tachycardia and tachypnea were defined based on World Health Organization (WHO) definitions [21]. Septic shock was defined by the presence of hypotension with end-organ dysfunction unresponsive to fluid replacement. Chest radiography was only performed if respiratory symptoms or signs were present, either on admission, or whenever they developed during the hospital stay. All lung involvement (pneumonia) was radiologically confirmed. Melioidosis-appropriate antibiotics were defined as the use of either ceftazidime, a carbapenem, trimethoprim-sulfamethoxazole, amoxicillin-clavulanate or doxycycline.

### Microbiological methods

Blood samples collected from patients were subjected to the BACTEC blood culture system according to the manufacturer’s instructions (Becton Dickinson, USA). Positive growth was subcultured onto blood agar, chocolate agar and MacConkey agar. Samples from other sources (pus, sputum, endotracheal secretions, pleural fluid) were cultured directly on blood agar, chocolate agar and MacConkey agar. *B*. *pseudomallei* was initially identified with either API20NE (BioMérieux, France) or BBL Crystal Identification Systems (Becton Dickinson, USA), and confirmed with real-time PCR targeting TTS1, as described previously [22]. Antibiotic susceptibility of *B*. *pseudomallei* isolates were determined by the Kirby-Bauer disk diffusion susceptibility test (Becton Dickinson, USA) and/or E-tests (BioMérieux, France). Multilocus sequence typing (MLST) was performed as described previously [23]. The presence of nucleotide sequences encoding *Burkholderia* intracellular motility factor A (BimA_Bm_ or BimA_Bp_) and filamentous hemagglutination genes (FhaB3), and the *Burkholderia thailandensis*-like flagellum and chemotaxis (BTFC) and the *Yersinia*-like fimbrial (YLF) gene clusters were determined using previously published methods [24].

### Statistical analysis

Statistical analysis was performed using SPSS Statistics 21. The Mann-Whitney *U* test was used for numerical variables and either the χ^2^ test or Fisher exact test was used for categorical variables. The correlation between the number of culture-confirmed melioidosis cases and average monthly rainfall in Central Sarawak was examined using Spearman’s rank co-efficient test. Population data were obtained from the Malaysian Census Data 2010. Meteorological data were obtained from the Malaysian Meteorological Department.

### Ethics statement

The study was approved by the Malaysian Medical Research Ethics Committee (NMRR-10-907-6862). Written informed consent was obtained from the parent or guardian of each child at enrollment. All retrospective data analyzed were anonymized.

## Results

### Epidemiological characteristics

**Fig 1:**
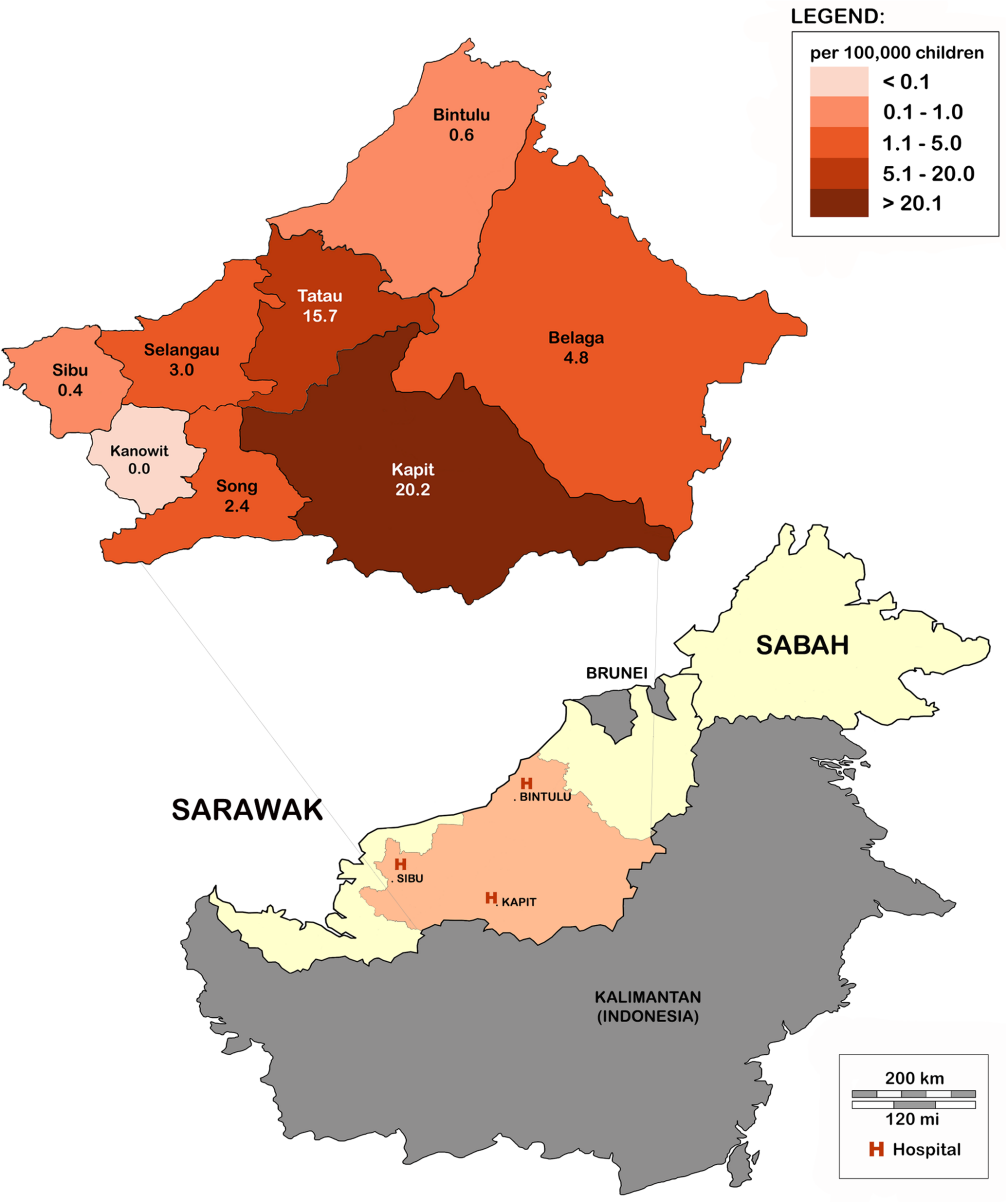
Average annual incidence of pediatric melioidosis by district within the Central region of Sarawak. The map of Borneo shows the Malaysian states of Sarawak and Sabah, and the location of the 3 study sites. The map insert depicts the average annual incidences of pediatric melioidosis in each district in Central Sarawak. The incidence per 100,000 children <15 years/ year, in each district, is labelled.

**Fig 2:**
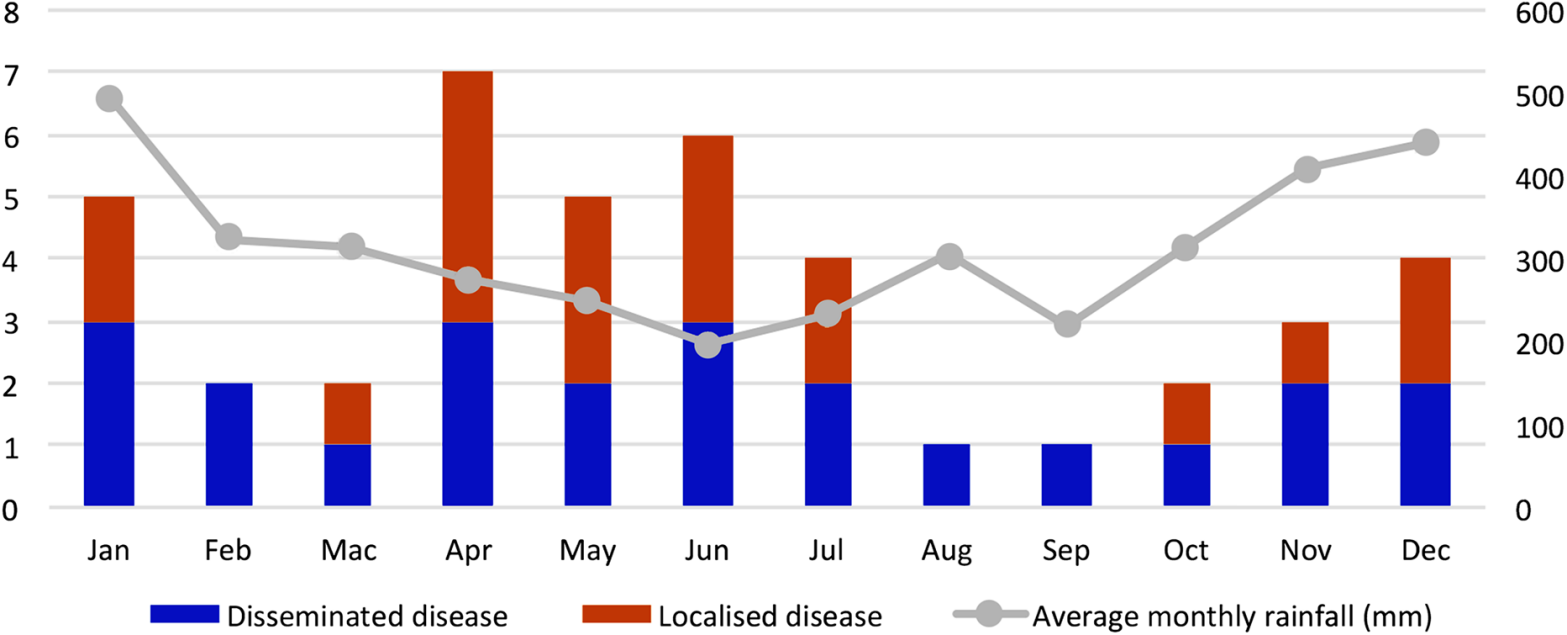
Distribution of the 42 pediatric melioidosis cases and average rainfall by month. The bar chart shows the distribution of the 42 pediatric melioidosis cases according to the month of admission. The average monthly rainfall over the 6-year period (January 2009—December 2014) in Central Sarawak is shown in the line graph. Cases are categorized into disseminated and localized melioidosis.

Forty-two children with culture-confirmed melioidosis were identified during the 6-year study period (3 in 2009, 5 in 2010, 12 in 2011, 8 in 2012, 9 in 2013 and 5 in 2014). The overall average annual incidence rate was 4.1 per 100,000 children <15 years, with marked variation between districts ranging from 0 to 20.2 per 100,000 children <15 years (Fig 1). Although more than 50% of children presented during the drier months of April to July (Fig 2), no significant correlation was found between the monthly incidence and average monthly rainfall (*r*_*s*_ = -0.078, *P* = 0.81). Of the 41 children with data available (S1 Fig), 33 (80%), including 9 of 10 fatal cases, resided in traditional longhouses in rural areas, where gravity-fed water systems supply homes with untreated water from streams.

### Clinical characteristics

#### Patient demographics and disease manifestations

Of the 42 children, 21 (50%) were <5 years, including 2 neonates. None had pre-existing medical conditions, although 13 (32%) had poor nutritional status. Twenty-three (55%) children had disseminated, and 19 (45%) had localized disease. The median duration of symptoms prior to admission was 14 (IQR 7–21) days. Most children had a history of fever at home, although 7 (17%), including 5 with localized disease, did not have a reported fever. Children with disseminated disease presented predominantly with respiratory and abdominal symptoms. They were unwell, had respiratory compromise and shock, lower lymphocyte and platelet counts, and abnormal blood biochemistry on admission (Table 1).

**Table 1:** Demographic, presenting clinical features on admission, subsequent findings and outcome of 42 children with culture-confirmed melioidosis.

Characteristic	All patients	Disseminated disease	Localized disease	*P*
No. of patients	42	23	19	
Demography				
Age, years, median (IQR)	4.7 (1.9–10.7)	2.7 (1.6–10.6)	6.3 (1.9–11.1)	0.29
Male sex	21 (50)	14 (61)	7 (37)	0.22
Iban ethnicity	37 (88)	21 (91)	16 (84)	0.64
Pre-existing medical condition	0 (0)	0(0)	0 (0)	-
Poor nutritional status†	13 (32)	9 (41)	4 (21)	0.31
Presenting symptoms				
Time between onset and admission, days, median (IQR)	14 (7–21)	12 (4–21)	14 (7–21)	0.24
Fever	35 (83)	21 (91)	14 (74)	0.21
Poor appetite	22 (52)	17 (74)	5 (26)	0.006
Head, neck or body swellings	21 (50)	4 (17)	17 (89)	<0.001
Cough	18 (43)	15 (65)	3 (16)	0.004
Lethargy or irritability	16 (38)	15 (65)	1 (5)	<0.001
Chills or rigors	16 (38)	10 (43)	6 (32)	0.64
Vomiting or diarrhea	15 (36)	15 (65)	0 (0)	<0.001
Shortness of breath	13 (31)	12 (52)	1 (5)	0.003
Abdominal pain*	6 (22)	5 (38)	1 (7)	0.08
URTI symptoms	5 (12)	3 (13)	2 (11)	1.00
Headache*	4 (15)	3 (23)	1 (7)	0.33
Presenting signs				
Tachycardia for age	27 (64)	20 (87)	7 (37)	0.002
Tachypnea for age	25 (60)	20 (87)	5 (26)	<0.001
Looks unwell	18 (43)	17 (74)	1 (5)	<0.001
Signs of respiratory distress	16 (38)	15 (65)	1 (5)	<0.001
Hepatomegaly	15 (36)	14 (61)	1 (5)	0.001
Lymph node swelling	13 (31)	3 (13)	10 (53)	0.02
Signs of dehydration	12 (29)	11 (48)	1 (5)	0.007
Abnormal lung findings	12 (29)	10 (43)	2 (11)	0.04
Pallor	11 (26)	9 (39)	2 (11)	0.08
Hypoxemia	10 (24)	10 (43)	0 (0)	0.001
Splenomegaly	9 (21)	9 (39)	0 (0)	0.002
Poor perfusion	9 (21)	9 (39)	0 (0)	0.002
Investigations^§^				
Hemoglobin, g/dL, median (IQR)	10.6 (9.5–11.6)	10.2 (9.4–11.4)	10.7 (9.7–12.3)	0.43
WBC, X 10^9^ cells/L, median (IQR)	11.8 (8.2–16.5)	9.9 (5.8–12.7)	13.6 (11.4–16.6)	0.03
Neut, X 10^9^ cells/L, median (IQR)	9.4 (5.3–11.6)	8.6 (4.0–14.4)	9.5 (8.5–11.6)	0.42
Lymph, X 10^9^ cells/L, median (IQR)	2.3 (1.2–3.8)	1.9 (0.6–2.3)	3.2 (2.4–5.5)	0.005
Platelet count, X 10^9^/L, median (IQR)	386 (243–498)	279 (172–414)	454 (382–612)	<0.001
ESR, mm/hr, median (IQR)	70 (50–100)	79 (51–98)	66 (49–107)	0.69
Sodium, mmol/L, median (IQR)	134 (128–136)	128 (126–134)	136 (135–138)	<0.001
Urea, mmol/L, median (IQR)	3.2 (2.3–5.2)	4.5 (2.8–6.5)	2.7 (2.1–3.3)	0.004
Creatinine, μmol/L, median (IQR)	39 (29–56)	54 (36–96)	33 (25–40)	0.03
Albumin, g/L, median (IQR)	28 (25–34)	25 (23–28)	37 (29–44)	<0.001
AST, U/L, median (IQR)	81 (36–261)	132 (46–359)	25 (19–40)	0.001
ALT, U/L median (IQR)	32 (19–133)	59 (22–145)	19 (12–52)	0.06
Subsequent findings/ outcome				
Bacteremia	20 (48)	20 (87)	0 (0)	<0.001
Septic shock	13 (31)	13 (57)	0 (0)	<0.001
Died	10 (24)	10 (43)	0 (0)	0.001

Data are No. (%) unless otherwise indicated.

† 41 children had nutritional status data available for analysis (disseminated disease, n = 22; localized disease, n = 19).

* 27 children (≥ 30 months) who could explicitly indicate if they were in pain (disseminated disease, n = 13; localized disease, n = 14).

^§^ Only investigations done on the day of admission were included: 41 children had Hemoglobin, WBC, Platelet count (disseminated disease, n = 23; localized disease, n = 18); 27 children had differential counts (disseminated disease, n = 16; localized disease, n = 11); 27 children had ESR (disseminated disease, n = 14; localized disease, n = 13); 39 children had Sodium, Urea (disseminated disease, n = 22; localized disease, n = 17); 24 children had Creatinine (disseminated disease, n = 14; localized disease, n = 10), 24 children had Albumin (disseminated disease, n = 17; localized disease, n = 7); 25 children had AST, ALT (disseminated disease, n = 18; localized disease, n = 7).

Abbreviations: IQR, interquartile range; WBC, white blood cell count; Neut, neutrophil count; Lymph, lymphocyte count; ESR, erythrocyte sedimentary rate; AST, aspartate aminotransferase; ALT, alanine aminotransferase.

Ten (24%) children died; all had disseminated disease (*P* = 0.001) and bacteremia (*P*<0.001). The case fatality rate increased with the number of affected organ sites (Table 2). Septic shock was strongly associated with mortality, noted in all (100%) deaths but in only 3 of 32 (9%) survivors (*P*<0.001). The case fatality rate was 77% (10/13) when septic shock was present.

**Table 2:** Distribution of the 42 culture-confirmed pediatric melioidosis cases according to the number of affected organ sites.

Number of affected organ sites	Number of patients	% of all cases	Number of deaths	% who died
0 (bacteremia only)	1	2	0	0
1	28	67	4	14
2	9	21	3	33
3	4	10	3	75

The commonest site of infection was the lungs, occurring in 21 (50%) children (Table 3). Eighteen (86%) of these children were bacteremic, including all 6 children whose lungs were involved as a secondary infection site. Children whose lungs were involved as a secondary site had a statistical trend for higher fatality than children whose lungs were the primary infection site (5/6 [83%] vs 5/15 [33%], *P* = 0.06, OR 10.0, 95%CI 0.9–110.3). When the lungs were the primary infection site, chest radiographs in all except 1 of the children showed unilateral, lobar consolidation. The remaining child had right peri-hilar haziness. Para-pneumonic effusion was observed in two children. In comparison, when the lungs were a secondary infection site, chest radiographs invariably showed bilateral, widespread, nodular or patchy alveolar opacities.

**Table 3:** Primary and secondary infection sites in 42 cases of culture-confirmed pediatric melioidosis.

	Number of patients	% of all cases	Number of deaths	% who died
Primary site				
Disseminated disease				
Lung	13	31	5	38
No evident focus	7	17	4	57
Lymph node	3	7	1	33
Total	23	55	10	43
Localized disease				
Lymph node	10	24	0	0
Skin/ soft tissue	3	7	0	0
Eye	3	7	0	0
Lung	2	5	0	0
Parotid	1	2	0	0
Total	19	45	0	0
Secondary site				
Spleen	8	19	3	38
Lung	6	14	5	83
Joint	4	10	2	50
Brain†	2	5	2	100
Muscle	1	2	0	0
Eye	1	2	1	100
Liver	1	2	1	100
Total*	23 (in 15 patients)	36	7	47

† Both patients were encephalopathic on admission, but had no focal neurological findings. 1 had abnormal cerebrospinal fluid examination, while the other had no lumbar puncture done but progressed to brain death.

* Some children had more than 1 secondary infection site.

Seven (17%) children presented initially with undifferentiated fever, and no evident infection focus. Six (86%) subsequently developed ≥1 secondary foci, mainly affecting the lungs (83%) and joints (50%). Children with no evident infection focus had a higher fatality rate when compared with children who had an overt primary infection site (4/7 [57%] vs 6/35 [17%], *P* = 0.04, OR 6.4, 95%CI 1.1–36.6). Among children with disseminated disease, the median duration of illness prior to admission was significantly longer in those with no evident infection focus than in those who had an overt primary infection site [15 (IQR 14–24) vs 7 (IQR 4–19) days, *P* = 0.03].

Lymph node infection occurred in 13 (31%) children, and was the commonest presentation in those with localized disease. Only 1 child with lymph node involvement was bacteremic, and had fatal disease. Ten children had unilateral cervical node infection, with either a single, or multiple node enlargement. Only one child had bilateral cervical involvement. The cervical node infections manifested as painless adenopathy (n = 3), lymphadenitis (n = 4) or lymph node abscesses (n = 4). Two children had unilateral axillary nodal abscesses; both had histories suggestive of recent skin or soft tissue infection at the lymphatic drainage area. Notably, only 1 child had melioidosis parotitis.

The spleen was the commonest intra-abdominal organ involved; occult splenic abscesses were found in 8 (53%) of the 15 children who had abdominal ultrasound examination. These lesions were invariably multiple, and measured <1cm. In contrast, liver abscesses were seen on ultrasound in only 1 (7%) child.

Other common sites of infection were the joints and the eyes. Joints were invariably secondary infection sites; 2 (50%) children had multiple septic joints while the remaining 2 had mono-arthritis. Knee (n = 3) and ankle (n = 2) joints were most commonly affected. Eye involvement was confined to unilateral lacrimal gland infection, presenting as an inflamed tender swelling at the medial aspect of the lower eyelid (n = 4).

There were 2 cases of neonatal melioidosis, both with bacteremia. The first was an asymptomatic, 2-day old infant who was admitted and investigated for possible early-onset neonatal sepsis, when his puerperal mother developed fever following his birth. The second case, a 9-day old infant, was admitted for late-onset neonatal sepsis when he presented with fever without a focus. Detailed history revealed that he was bathed, and his milk bottles washed, using unchlorinated surface water.

#### Case management

Data on primary care management were available in 29 children, including 9 of the 10 fatal cases (S1 Fig). Twenty-five (86%) had ambulatory care prior to hospital admission, without the diagnosis of melioidosis being suspected. These children were mainly diagnosed to have upper respiratory tract infections, viral fevers, non-specific fevers or neck adenitis. All but one (including the 9 fatal cases) received oral antibiotics; none received the melioidosis-appropriate antibiotics trimethoprim-sulfamethoxazole, amoxicillin-clavulanate or doxycycline.

In hospital, all 10 fatal cases received ventilatory, inotropic and intensive care support; 7 (70%) of whom required intubation within 6 hours of hospital admission. All 10 received melioidosis-appropriate antibiotics (ceftazidime or a carbapenem); 8 (80%) had treatment initiated immediately on admission. Deaths occurred within a median of 2 (IQR 1–6) days following admission. Among survivors, fever resolved completely within a median of 4 (IQR 1–8) days after initiation of melioidosis-appropriate antibiotics. All survivors received oral eradication therapy with a combination of trimethoprim-sulfamethoxazole and amoxicillin-clavulanate for 20 weeks duration. There were no relapses recorded during the study period.

Only 1 child received gentamicin at any time during the course of the illness. This was the asymptomatic 2-day old infant (mentioned above), who was given parenteral penicillin and gentamicin for 2 days before his blood culture results (*B*. *pseudomallei)* were known. His antibiotics were subsequently switched to ceftazidime.

### Microbiological characteristics

*B*. *pseudomallei* was isolated from a single specimen in 39 children, from 2 different specimen types in 2 children, and from 3 different specimen types in 1 child. Blood was the commonest source of a positive isolate (n = 20), followed by pus (n = 19), endotracheal aspirates (n = 4), sputum (n = 2) and pleural fluid (n = 1). No isolates were detected from other non-sterile site specimens.

*B*. *pseudomallei* isolates from 36 patients were tested for gentamicin susceptibility (S1 Fig); 35 (97%) were susceptible to gentamicin (20 by disc diffusion method, 5 by E-test [MIC 0.5–1.5 μg/ml], and 10 by both methods) (S1 Table). Eleven of these clinical isolates, representative of the aforementioned varied epidemiological and clinical findings, were available for further molecular characterization (Table 4). All 11 (100%) isolates were resolved into a single sequence type, ST881, and possessed the *bimA*_Bp_ variant of *bimA*, *fhaB3*, and the YLF gene cluster.

**Table 4:** Clinical and microbiological features of 11 patients whose *B*. *pseudomallei* isolates were available for molecular characterization.

No.	Age	Date admitted	District	Disseminated disease	Outcome	Source of isolate	Susceptible to Gentamicin	MLST	Virulence determinants present
1	3y 1m	Oct 2010	Kapit	No	Survived	Pus	Yes	ST881	*bimA*_Bp_, *fhaB3*, YLF
2	2y 2m	Mar 2011	Kapit	Yes	Died	Blood	Yes	ST881	*bimA*_Bp_, *fhaB3*, YLF
3	2y 6m	Mar 2011	Tatau	No	Survived	Pus	Yes	ST881	*bimA*_Bp_, *fhaB3*, YLF
4	3y 4m	Apr 2011	Kapit	No	Survived	Pus	Yes	ST881	*bimA*_Bp_, *fhaB3*, YLF
5	11y 1m	Apr 2011	Selangau	No	Survived	Pus	Yes	ST881	*bimA*_Bp_, *fhaB3*, YLF
6	13y 4m	May 2011	Kapit	Yes	Survived	Pus	Yes	ST881	*bimA*_Bp_, *fhaB3*, YLF
7	11y 10m	Jun 2011	Kapit	No	Survived	Pus	Yes	ST881	*bimA*_Bp_, *fhaB3*, YLF
8	1y 8m	Jul 2011	Bintulu	No	Survived	Pus	Yes	ST881	*bimA*_Bp_, *fhaB3*, YLF
9	8y 4m	Nov 2011	Tatau	Yes	Died	Blood	Yes	ST881	*bimA*_Bp_, *fhaB3*, YLF
10	12y 10m	Dec 2011	Selangau	No	Survived	Pus	Yes	ST881	*bimA*_Bp_, *fhaB3*, YLF
11	3y	Dec 2011	Kapit	Yes	Survived	Blood	Yes	ST881	*bimA*_Bp_, *fhaB3*, YLF

Abbreviations: y, years; m, months

## Discussion

This study is the first detailed report of childhood melioidosis in Sarawak, and shows that *B*. *pseudomallei* infections in this region have important epidemiological, clinical and microbiological differences when compared with other melioidosis endemic regions.

The incidence rate of pediatric melioidosis in Central Sarawak (4.1 per 100,000 children), and in particular Kapit and Tatau districts (20.2 and 15.7 per 100,000 children respectively), were among the highest observed in any melioidosis endemic region, including the Malaysian states of Sabah and Pahang (0.64 and 0.68 per 100,000 children respectively) and northern Australia (5.48 per 100,000 children) [13, 14, 25]. A large study in Cambodia has also recently reported extremely high pediatric melioidosis incidences (28–35 cases per 100,000 children) [26]. In fact, the pediatric incidence rates recorded in this Cambodian study, and in the present one, are comparable to that of adult cases in northern Australia (19.6/ 100,000) and northeast Thailand (21.3/ 100,000) [27, 28], highlighting the significant and likely unrecognized burden of melioidosis in children. This underscores an urgent need to better define the global and regional epidemiology of pediatric melioidosis, as well as strengthen diagnostic capacities. Despite our findings, we believe the incidence of pediatric melioidosis in Central Sarawak remains under-estimated. Firstly, bacterial culture, the gold standard for diagnosis, lacks sensitivity [29], and may only be positive after repeated attempts. This is exemplified in Patient 9 (Table 4), who, having had negative admission blood cultures despite a prolonged illness of 14 days, developed septic shock, and succumbed 72 hours later. *B*. *pseudomallei* was only isolated from a repeat blood culture obtained shortly before she died. Second, a culture-confirmed diagnosis may have been missed in a number of patients in Central Sarawak, as selective media were not used in the study. *B*. *pseudomallei*, a slow growing bacillus, is often rapidly overgrown by the normal flora when samples from non-sterile sites are cultured using routine bacterial agar such as blood or MacConkey agar. *B*. *pseudomallei* selective media such as Ashdown media are known to increase the yield of *B*. *pseudomallei* from these non-sterile sites. Ashdown media, however, contains gentamicin, and is unsuitable for use in Central Sarawak. Alternative selective media, for instance one that utilizes colistin instead of gentamicin, should be considered, and may further enhance the detection of melioidosis cases in this region.

Another interesting finding was the marked regional variation in incidences, both in Sarawak as a whole, and within Central Sarawak. A review of the microbiology laboratory records of all major public hospitals outside of Central Sarawak revealed only 3 culture-confirmed pediatric melioidosis cases during the entire study period. In addition, a similar epidemiology is also seen in the adult population, with high melioidosis incidences confined to Central Sarawak (Personal communication, Dr. Jeffery Anak Stephen, Sarawak State Health Department, 1^st^ April 2017). Within Central Sarawak, incidences varied widely between the different districts, despite all 3 study centers using similar diagnostic approaches. Indeed, differences in diagnostics or healthcare seeking behavior are unlikely to explain the apparent ‘hotspots’ in the adjacent districts of Kapit and Tatau, as children in Kapit district are seen in Kapit Hospital whereas those in Tatau district are seen in Bintulu Hospital (S2 Table). Thus, we believe these ‘hotspots’ of higher melioidosis incidence to be real, and not artefactual. Interestingly, a similar finding of regional ‘hotspots’ was also noted in the recent Cambodian study [26]. The reasons for these regional variations in incidences remain unknown, but may be related to differences in the local environment or in human behavioral activities and living conditions. However, there are very few differences in the behavioral activities and living conditions present throughout Sarawak, possibly indicating the existence of true ecological hotspots in Central Sarawak. Future environmental studies will be crucial in confirming these hypotheses.

Pediatric melioidosis infections in Sarawak did not appear to be related to rainfall, in contrast to the well-established melioidosis epidemiology in other regions [3]. Although more than half the cases were admitted during the drier months of our study, there was no statistically significant correlation between the monthly incidence and the average monthly rainfall, possibly due to the small sample size and short study period. The observed higher number of cases during the drier months may be related to the various water sources from which rural households obtain their water supply. In Central Sarawak, the primary water source for rural households are gravity-fed systems that draw water from higher-elevated streams. During the dry season, these systems are often disrupted. Households are then forced to seek alternate water sources, mainly rivers and ponds, for both bathing and drinking. These water sources and its surrounding environments may be important reservoirs of *B*. *pseudomallei*. Similar environmental reservoirs have been found in relation to pediatric melioidosis infections in Balimo, Papua New Guinea [30].

Previously published studies have shown that the majority of children with melioidosis present with localized disease [11, 26]. However, in our study, 55% of children had disseminated disease. All deaths occurred in this disseminated disease group, with a case fatality rate of 43%. This pattern has also been noted in other studies. For example, in Cambodia, deaths occurred in 72% of bacteremic and in <1% of non-bacteremic children [26]. The overall mortality rate was 24% in the present study, in comparison to 7–22% in northern Australia, Thailand and Cambodia [8, 11, 26]. Despite the high mortality seen in Central Sarawak, melioidosis remains largely neglected when compared with other infectious diseases such as dengue, receiving little attention from both the local media and health authorities. Of note, dengue infection, commonly considered the most important infectious disease in Malaysia, did not cause any deaths in children <15 years in Central Sarawak during the study period (Personal communication, Dr. Jeffery Anak Stephen, Sarawak State Health Department, 1^st^ April 2017).

In adults, melioidosis is considered an opportunistic infection; risk factors including diabetes mellitus, renal impairment, hazardous alcohol use and immunosuppression are identified in as many as 80% of patients [31]. In children, the importance of host risk factors remain undetermined. None of the children in our study had any apparent predisposing medical conditions, a finding that was also observed in Pahang [13]. Similarly, only 3% of children in the Cambodian study had significant co-morbidity [26]. In contrast, 41% of children with melioidosis in Sabah (another Malaysian Bornean state), the majority of whom were ethnic Kadazandusuns, had β-thalassemia major, while 31% of children in Kuala Lumpur (in peninsular Malaysia) had hematological malignancies [14, 32]. Five (12%) children in our study had moderate or severe anemia at presentation (hemoglobin 6.4–7.8 g/dL). Although there were no red blood cell indices or blood films available, the likelihood that these anemias were due to β-thalassemia is low. β-thalassemia major and intermedia have never been reported in the ethnic Iban population of Sarawak [33]. The ethnic Ibans, who make up the majority of patients in our study, do not possess the β-globin gene deletion which is found in 13% of the ethnic Kadazandusuns of neighboring Sabah [34]. A limitation of the present study, however, was that patients were not investigated for α-thalassemia, as well as other occult inherited conditions, including primary immunodeficiency disorders, which may possibly predispose to melioidosis [35]. Nearly 1/3 did have poor nutritional status, as did 13% and 48% of children in northern Australia and Cambodia respectively [8, 26]. Malnutrition may thus be an important risk factor in pediatric melioidosis, and warrants further investigation.

Pneumonia and undifferentiated fever were the two most important manifestations of disseminated disease in Sarawak. Pneumonia, the commonest manifestation, was found in 83% of children with disseminated disease. In 1/3 of these children, the lungs had been infected as a secondary site. Although limited by a small sample size, our analysis indicates a statistical trend towards a higher risk of death in these children who had lung involvement as a secondary site. We postulate that this finding is related to increased disease severity from an extensive hematogenous dissemination that is occurring when the lungs are involved as a secondary site, evidenced by the universal presence of bacteremia and bilateral, widespread, alveolar opacities on the chest radiographs. Undifferentiated fever was the next commonest presentation of disseminated melioidosis. The high fatality rate seen with this presentation may be related to the more prolonged, untreated, disease observed in these children.

Children with localized melioidosis in Sarawak presented primarily with cervical node swelling, subcutaneous abscesses or lacrimal gland infections. These presentations highlight the intriguing regional differences in clinical manifestations of childhood melioidosis. Lacrimal gland infection, or dacrocystitis, caused by *B*. *pseudomallei* has not previously been reported. In contrast, cutaneous melioidosis and suppurative parotitis are the predominant manifestations of localized melioidosis in northern Australia and mainland Southeast Asia respectively [8, 36]. Further studies are needed to elucidate the reasons for these regional variations. Ingestion of *B*. *pseudomallei* from contaminated unchlorinated water has been suggested as a cause of parotitis in Southeast Asia [37], and may well underlie the cervical node infections seen in Sarawak. Nevertheless, other regional epidemiological and bacterial virulence differences may also be important.

A delay in seeking medical attention and/or clinical diagnosis was observed in most patients in the study, and was a major contributing factor to the high fatality rate. These fatalities occurred soon after admission despite intensive care support. In northern Australia, melioidosis fatalities reduced from 30% to 9% mainly through earlier diagnosis, earlier treatment, and improved access to intensive care [31]. Thus, in Sarawak, increasing the awareness in the local community and in primary healthcare providers will also be key to improving outcomes. Melioidosis must be considered in children who have pneumonia, prolonged fever, cervical lymph node swelling or septic arthritis. In addition, the empiric antibiotics recommended for these common pediatric conditions, based on guidelines developed primarily in non-melioidosis endemic regions, may not be appropriate. For example, amoxicillin or benzyl penicillin is recommended for children with community-acquired pneumonia, while cloxacillin is recommended for empirical treatment in septic arthritis [38]. As these antibiotics have no activity against *B*. *pseudomallei*, empirical treatment with ceftazidime should instead be considered, especially in a child with suspected melioidosis and severe sepsis. However, it is also important to note that ceftazidime (a broad spectrum third-generation cephalosporin) is a potent extended-spectrum beta-lactamase inducer [39], and injudicious use as an empirical antibiotic may lead to the development of antimicrobial resistance problems in hospitals. As such, determining the regional epidemiology of *B*. *pseudomallei* and improving the understanding of the disease manifestations remain crucial.

Ninety-seven percent (35/36) of *B*. *pseudomallei* isolates tested were susceptible to gentamicin. We have shown previously that gentamicin-susceptible *B*. *pseudomallei* isolates in Central Sarawak are of ST881, or its single-locus variant ST997 [17]. In the current study, we determined the presence of 3 putative virulence factors, namely BimA, FhaB3, and the BTFC and YLF gene clusters [24], to find possible explanations for the distinctive clinical characteristics observed in Central Sarawak. All *B*. *pseudomallei* strains possess BimA, which confers the bacteria’s ability to move between eukaryotic cells and evade host immune responses [40]. BimA consists of two variants, BimA_Bm_ and BimA_Bp_. A study in northern Australia showed that the *bimA*_Bm_ variant found in 12% of their isolates was associated with a 14 times higher risk of neurological melioidosis, while patients infected with the commoner *bimA*_Bp_ were twice as likely to develop pneumonia [24]. All isolates in the present study were of *bimA*_Bp_ variant. This is consistent with the rarity of neurological disease in Sarawakian children. FhaB3 is a variably present anti-macrophage factor that similarly renders *B*. *pseudomallei* strains the ability to evade host immune responses [41]. 83% of Australian isolates possessed *fhaB3*, with *fhaB3* positive patients twice as likely to be blood culture positive and *fhaB3* negative patients more likely to have localized cutaneous disease [24]. All isolates in the present study possessed *fhaB3*, which may explain the high rate of bacteremia and disseminated disease seen in the study and conversely, the rarity of cutaneous disease. BTFC and YLF gene clusters are mutually exclusive in the *B*. *pseudomallei* genome, with YLF previously shown to be over-represented in clinical isolates [42]. The YLF cluster is more common in Thailand, while 79% of Australian isolates possessed the BTFC cluster, although no correlation with any clinical presentation has yet been found [24]. All isolates in the present study possessed the YLF cluster.

In conclusion, Central Sarawak has a very high incidence of pediatric melioidosis, caused predominantly by gentamicin-susceptible *B*. *pseudomallei* strains. Children frequently presented with disseminated disease and had an alarmingly high death rate, despite the absence of any apparent predisposing risk factor. Enhancing awareness and recognition of the protean manifestations of melioidosis will hopefully lead to better patient care and outcomes.

## Supporting information

S1 Checklist STROBE checklist. (DOC)

S1 Fig Study flow diagram. (DOCX)

S1 Table Minimum inhibitory concentrations using E-test for *B*. *pseudomallei* isolates in this study. (DOC)

S2 Table Characteristics of the pediatric melioidosis cases according to study site and catchment district. (DOCX)

## References

[1] Wiersinga WJ, Currie BJ, Peacock SJ. Melioidosis. N Engl J Med. 2012 9 13;367(11):1035–44. doi: 10.1056/NEJMra1204699 22970946 10.1056/NEJMra1204699

[2] Strauss JM, Groves MG, Mariappan M, Ellison DW. Melioidosis in Malaysia. II. Distribution of Pseudomonas pseudomallei in soil and surface water. Am J Trop Med Hyg. 1969;18:698–702. 5810797

[3] Cheng AC, Currie BJ. Melioidosis: epidemiology, pathophysiology, and management. Clin Microbiol Rev. 2005 4;18(2):383–416. doi: 10.1128/CMR.18.2.383-416.2005 15831829 10.1128/CMR.18.2.383-416.2005 PMC1082802

[4] Chaowagul W, White NJ, Dance DA, Wattanagoon Y, Naigowit P, Davis TM, et al Melioidosis: a major cause of community-acquired septicemia in northeastern Thailand. J Infect Dis. 1989;159:890–9. 2708842 10.1093/infdis/159.5.890

[5] Limmathurotsakul D, Golding N, Dance DA, Messina JP, Pigott DM, Moyes CL, et al Predicted global distribution of Burkholderia pseudomallei and burden of melioidosis. Nature microbiology. 2016 1 11;1:15008 doi: 10.1038/nmicrobiol.2015.8 27571754 10.1038/nmicrobiol.2015.8

[6] Domthong P, Chaisuksant S, Sawanyawisuth K. What clinical factors are associated with mortality in septicemic melioidosis? A report from an endemic area. J Infect Dev Ctries. 2016 4 28;10(4):404–9. doi: 10.3855/jidc.6455 27131004 10.3855/jidc.6455

[7] Sanderson C, Currie BJ. Melioidosis: a pediatric disease. Pediatr Infect Dis J. 2014 7;33(7):770–1. doi: 10.1097/INF.0000000000000358 24732448 10.1097/INF.0000000000000358

[8] McLeod C, Morris PS, Bauert PA, Kilburn CJ, Ward LM, Baird RW, et al Clinical presentation and medical management of melioidosis in children: a 24-year prospective study in the Northern Territory of Australia and review of the literature. Clin Infect Dis. 2015 1 1;60(1):21–6. doi: 10.1093/cid/ciu733 25228703 10.1093/cid/ciu733

[9] Thatrimontrichai A, Maneenil G. Neonatal melioidosis: systematic review of the literature. Pediatr Infect Dis J. 2012 11;31(11):1195–7. doi: 10.1097/INF.0b013e318265ac62 22739573 10.1097/INF.0b013e318265ac62

[10] Ralph A, McBride J, Currie BJ. Transmission of Burkholderia Pseudomallei Via Breast Milk in Northern Australia. Pediatr Infect Dis J. 2004 12;23(12):1169–71. . 15626961

[11] Lumbiganon P, Viengnondha S. Clinical manifestations of melioidosis in children. Pediatr Infect Dis J. 1995;14(2):136–40. 7746696 10.1097/00006454-199502000-00010

[12] Kandasamy Y, Norton R. Paediatric melioidosis in North Queensland, Australia. J Paediatr Child Health. 2008 12;44(12):706–8. doi: 10.1111/j.1440-1754.2008.01410.x 19054292 10.1111/j.1440-1754.2008.01410.x

[13] How HS, Ng KH, Yeo HB, Tee HP, Shah A. Pediatric melioidosis in Pahang, Malaysia. J Microbiol Immunol Infect. 2005 10;38(5):314–9. . 16211138

[14] Fong SM, Wong KJ, Fukushima M, Yeo TW. Thalassemia major is a major risk factor for pediatric melioidosis in Kota Kinabalu, Sabah, Malaysia. Clin Infect Dis. 2015 6 15;60(12):1802–7. doi: 10.1093/cid/civ189 25767257 10.1093/cid/civ189

[15] Embi N, Suhaimi A, Mohamed R, Ismail G. Prevalence of antibodies to Pseudomonas pseudomallei exotoxin and whole cell antigens in military personnel in Sabah and Sarawak, Malaysia. Microbiol Immunol. 1992;36(8):899–904. . 1474938 10.1111/j.1348-0421.1992.tb02092.x

[16] Yew KL, Ng TH, How SH, Kuan YC. The bug and the big heart—melioidotic pericardial effusion. Med J Malaysia. 2011 3;66(1):71–2. . 23765151

[17] Podin Y, Sarovich DS, Price EP, Kaestli M, Mayo M, Hii K, et al Burkholderia pseudomallei Isolates from Sarawak, Malaysian Borneo, Are Predominantly Susceptible to Aminoglycosides and Macrolides. Antimicrob Agents Chemother. 2014 1;58(1):162–6. doi: 10.1128/AAC.01842-13 24145517 10.1128/AAC.01842-13 PMC3910780

[18] McEniry DW, Gillespie SH, Felmingham D. Susceptibility of Pseudomonas pseudomallei to new beta-lactam and aminoglycoside antibiotics. J Antimicrob Chemother. 1988;21:171–5. 3162898 10.1093/jac/21.2.171

[19] WHO. WHO Child Growth Standards: WHO Press; [30 3 2017]. Available from: http://www.who.int/childgrowth/en/.

[20] Wang Y, Chen H. Use of Percentiles and Z-Scores in Anthropometry In: Preedy VR, editor. Handbook of Anthropometry: Physical Measures of Human Form in Health and Disease. New York, USA: Springer; 2012 p. 29–48.

[21] WHO. Pocket Book of Hospital care for children: Guideline for the management of common illnesses with limited resources. Geneva, Switzerland: WHO Press; 2005 p. 378.

[22] Novak RT, Glass MB, Gee JE, Gal D, Mayo MJ, Currie BJ, et al Development and Evaluation of a Real-Time PCR Assay Targeting the Type III Secretion System of Burkholderia pseudomallei. J Clin Microbiol. 2006 1;44(1):85–90. doi: 10.1128/JCM.44.1.85-90.2006 16390953 10.1128/JCM.44.1.85-90.2006 PMC1351940

[23] Godoy D, Randle G, Simpson AJ, Aanensen DM, Pitt TL, Kinoshita R, et al Multilocus sequence typing and evolutionary relationships among the causative agents of melioidosis and glanders, Burkholderia pseudomallei and Burkholderia mallei. J Clin Microbiol. 2003;41(5):2068–79. doi: 10.1128/JCM.41.5.2068-2079.2003 12734250 10.1128/JCM.41.5.2068-2079.2003 PMC154742

[24] Sarovich DS, Price EP, Webb JR, Ward LM, Voutsinos MY, Tuanyok A, et al Variable virulence factors in Burkholderia pseudomallei (melioidosis) associated with human disease. PLoS One. 2014;9(3):e91682 Pubmed Central PMCID: PMC3950250. doi: 10.1371/journal.pone.0091682 24618705 10.1371/journal.pone.0091682 PMC3950250

[25] Edmond K, Bauert P, Currie B. Paediatric melioidosis in the Northern Territory of Australia: An expanding clinical spectrum. J Paediatr Child Health. 2001;37(4):337–41. 11532051 10.1046/j.1440-1754.2001.00660.x

[26] Turner P, Kloprogge S, Miliya T, Soeng S, Tan P, Sar P, et al A retrospective analysis of melioidosis in Cambodian children, 2009–2013. BMC Infect Dis. 2016 11 21;16(1):688 Pubmed Central PMCID: PMC5117556. doi: 10.1186/s12879-016-2034-9 27871233 10.1186/s12879-016-2034-9 PMC5117556

[27] Currie BJ, Jacups SP, Cheng AC, Fisher DA, Anstey NM, Huffam SE, et al Melioidosis epidemiology and risk factors from a prospective whole-population study in northern Australia. Trop Med Int Health. 2004 11;9(11):1167–74. doi: 10.1111/j.1365-3156.2004.01328.x 15548312 10.1111/j.1365-3156.2004.01328.x

[28] Limmathurotsakul D, Wongratanacheewin S, Teerawattanasook N, Wongsuvan G, Chaisuksant S, Chetchotisakd P, et al Increasing incidence of human melioidosis in Northeast Thailand. Am J Trop Med Hyg. 2010 6;82(6):1113–7. Pubmed Central PMCID: 2877420. doi: 10.4269/ajtmh.2010.10-0038 20519609 10.4269/ajtmh.2010.10-0038 PMC2877420

[29] Limmathurotsakul D, Jamsen K, Arayawichanont A, Simpson JA, White LJ, Lee SJ, et al Defining the true sensitivity of culture for the diagnosis of melioidosis using bayesian latent class models. PLoS One. 2010;5(8):e12485 Pubmed Central PMCID: 2932979. doi: 10.1371/journal.pone.0012485 20830194 10.1371/journal.pone.0012485 PMC2932979

[30] Warner JM, Pelowa DB, Gal D, Rai G, Mayo M, Currie BJ, et al The epidemiology of melioidosis in the Balimo region of Papua New Guinea. Epidemiol Infect. 2008 7;136(7):965–71. doi: 10.1017/S0950268807009429 17714600 10.1017/S0950268807009429 PMC2870883

[31] Currie BJ, Ward L, Cheng AC. The epidemiology and clinical spectrum of melioidosis: 540 cases from the 20 year Darwin prospective study. PLoS Negl Trop Dis. 2010;4(11):e900 doi: 10.1371/journal.pntd.0000900 21152057 10.1371/journal.pntd.0000900 PMC2994918

[32] Sam IC, Puthucheary SD. Melioidosis in children from Kuala Lumpur, Malaysia. Ann Trop Paediatr. 2006 9;26(3):219–24. doi: 10.1179/146532806X120318 16925959 10.1179/146532806X120318

[33] Clinical Practice Guidelines: Management of Transfusion Dependent Thalassaemia. In: Malaysia MoH, editor. Malaysia: Ministry of Health Malaysia; 2009.

[34] Tan JA, Lee PC, Wee YC, Tan KL, Mahali NF, George E, et al High prevalence of alpha- and beta-thalassemia in the Kadazandusuns in East Malaysia: challenges in providing effective health care for an indigenous group. J Biomed Biotechnol. 2010;2010 Pubmed Central PMCID: PMC2943116. doi: 10.1155/2010/706872 20871816 10.1155/2010/706872 PMC2943116

[35] Lee PP, Lau YL. Endemic infections in Southeast Asia provide new insights to the phenotypic spectrum of primary immunodeficiency disorders. Asian Pac J Allergy Immunol. 2013 9;31(3):217–26. . 24053704

[36] Dance DA, Davis TM, Wattanagoon Y, Chaowagul W, Saiphan P, Looareesuwan S, et al Acute suppurative parotitis caused by Pseudomonas pseudomallei in children. J Infect Dis. 1989;159:654–60. 2926159 10.1093/infdis/159.4.654

[37] Currie BJ. Melioidosis: evolving concepts in epidemiology, pathogenesis, and treatment. Seminars in respiratory and critical care medicine. 2015 2;36(1):111–25. doi: 10.1055/s-0034-1398389 25643275 10.1055/s-0034-1398389

[38] Malaysia MoH. National Antibiotic Guideline. Malaysia: Ministry of Health Malaysia; 2008 p. 253.

[39] Paterson DL, Bonomo RA. Extended-spectrum beta-lactamases: a clinical update. Clin Microbiol Rev. 2005 10;18(4):657–86. Pubmed Central PMCID: PMC1265908. doi: 10.1128/CMR.18.4.657-686.2005 16223952 10.1128/CMR.18.4.657-686.2005 PMC1265908

[40] Stevens JM, Galyov EE, Stevens MP. Actin-dependent movement of bacterial pathogens. Nat Rev Microbiol. 2006 2;4(2):91–101. doi: 10.1038/nrmicro1320 16415925 10.1038/nrmicro1320

[41] Dowling AJ, Wilkinson PA, Holden MT, Quail MA, Bentley SD, Reger J, et al Genome-wide analysis reveals loci encoding anti-macrophage factors in the human pathogen Burkholderia pseudomallei K96243. PLoS One. 2010;5(12):e15693 Pubmed Central PMCID: 3008741. doi: 10.1371/journal.pone.0015693 21203527 10.1371/journal.pone.0015693 PMC3008741

[42] Tuanyok A, Auerbach RK, Brettin TS, Bruce DC, Munk AC, Detter JC, et al A horizontal gene transfer event defines two distinct groups within Burkholderia pseudomallei that have dissimilar geographic distributions. J Bacteriol. 2007 12;189(24):9044–9. doi: 10.1128/JB.01264-07 17933898 10.1128/JB.01264-07 PMC2168593

