# Supplementary material for: Pediatric melioidosis in Sarawak, Malaysia: Epidemiological, clinical and microbiological characteristics
Source: PLoS Negl Trop Dis. 2017 Jun 9;11(6):e0005650. doi: 10.1371/journal.pntd.0005650 (PMC5479590; doi:10.1371/journal.pntd.0005650)
Supplement: S1 Fig — (DOCX) [file pntd.0005650.s002.docx]

**S1 Figure. Study flow diagram.**

No. of cases with complete data available for analysis according to data type:

- Clinical data, n = 42
- Epidemiological data, n = 41
- Primary care management data, n = 29

No. of cases who had their isolates’ gentamicin susceptibility tested

n = 36

Prospective cases

(July 2010 - Dec 2014)

n = 36

No. of cases who had their isolates genotyped

n = 11

*n* = 11

Isolate genotyping not done, n = 25 (3 remaining retrospective cases, 22 prospective cases due to budgetary constraints)

No home address, n = 1 (retrospective case)

Incomplete information on prior treatment at primary care clinic, n = 13 (1 retrospective, 12 prospective cases)

Isolates’ gentamicin susceptibility not tested (gentamicin susceptibility was initially not routinely tested), n = 6 (3 retrospective, 3 prospective cases)

Retrospective cases

(Jan 2009 - Jun 2010)

n = 6

Total No. of cases

n = 42
