# Supplementary material for: Pediatric melioidosis in Sarawak, Malaysia: Epidemiological, clinical and microbiological characteristics
Source: PLoS Negl Trop Dis. 2017 Jun 9;11(6):e0005650. doi: 10.1371/journal.pntd.0005650 (PMC5479590; doi:10.1371/journal.pntd.0005650)
Supplement: S1 Table — (DOC) [file pntd.0005650.s003.doc]

**S1 Table. Minimum inhibitory concentrations using E-test for *B. pseudomallei* isolates in this study.**

| Patient Code | Hospital | Minimum inhibitory concentration (μg/ml) | | | | | |
| --- | --- | --- | --- | --- | --- | --- | --- |
|  |  | AMX | SXT | CAZ | DOX | GEN | MEM |
| 07 | Bintulu | 1.5 | 0.38 | 1.0 | 0.25 | 1.0 | 0.5 |
| 09 | Bintulu | 3 | 2 | 2 | 0.75 | 1.0 | 0.75 |
| 10 | Bintulu | 3 | 4 | 1.5 | 1.5 | 1.5 | 0.5 |
| 11 | Bintulu | 3 | 2 | 1.0 | 1.0 | 0.75 | 1 |
| 15 | Sibu | 3 | 1.5 | 2 | 0.5 | 1.5 | 0.5 |
| 20 | Kapit | 3 | 3 | 1.5 | 1.0 | 1.0 | 0.5 |
| 22 | Kapit | 3 | 2 | 2 | 0.75 | 1.0 | 0.75 |
| 25 | Kapit | 8 | 2 | 4 | 0.75 | 0.5 | 1.0 |
| 27 | Kapit | 1.5 | 2 | 1.0 | 0.5 | 0.5 | 0.5 |
| 28 | Kapit | 2 | 0.75 | 1.0 | 0.38 | 1.0 | 0.5 |
| 29 | Kapit | 6 | 4 | 3 | 1.5 | 1.0 | 0.75 |
| 30 | Kapit | 2 | 0.25 | 0.38 | 0.25 | 1.5 | 0.25 |
| 33 | Kapit | 2 | >4 | 1.0 | 0.25 | 0.75 | 0.75 |
| 37 | Sibu | 3 | 1.0 | 2 | 0.38 | 1.5 | 0.75 |
| 38 | Sibu | 3 | 2 | 2 | 0.5 | 1.5 | 1.0 |

Abbreviations: AMX, amoxicillin-clavulanic acid; CAZ, ceftazidime; DOX, doxycycline; GEN, gentamicin; MEM, meropenem; SXT, trimethoprim-sulfamethoxazole; ND, Not determined.
