# Supplementary material for: Pediatric melioidosis in Sarawak, Malaysia: Epidemiological, clinical and microbiological characteristics
Source: PLoS Negl Trop Dis. 2017 Jun 9;11(6):e0005650. doi: 10.1371/journal.pntd.0005650 (PMC5479590; doi:10.1371/journal.pntd.0005650)
Supplement: S2 Table — (DOCX) [file pntd.0005650.s004.docx]

**S2 Table. Characteristics of the pediatric melioidosis cases according to study site and catchment district.**

| Characteristics | Study sites | | | | | | | |
| --- | --- | --- | --- | --- | --- | --- | --- | --- |
|  | Kapit Hospital | | Bintulu Hospital | | | Sibu Hospital | | |
| Catchment district | Kapit | Song | Bintulu | Tatau | Belaga | Sibu | Selangau | Kanowit |
| Catchment population (aged <15 years) | 18,992 | 6,960 | 53,023 | 8,509 | 10,439 | 43,915 | 22,440 | 8,272 |
| No. of cases | 23 | 1 | 2 | 8 | 3 | 1 | 4 | 0 |
| Incidence rate (per 100, 000 aged <15 years) | 20.2 | 2.4 | 0.6 | 15.7 | 4.8 | 0.4 | 3.0 | 0.0 |
| No. of cases with disseminated disease (% of total no. of cases in district) | 11 (48) | 0 (0) | 1(50) | 5(63) | 2 (67) | 1(100) | 3 (75) | - |
| No. of cases with bacteremia (% of total no. of cases in district) | 9 (39) | 0 (0) | 1(50) | 5(63) | 2 (67) | 1(100) | 2 (50) | - |
| No. of cases who had shock [at any time] (% of total no. of cases in district) | 5 (22) | 0 (0) | 1(50) | 3 (38) | 2 (67) | 0 (0) | 2 (50) | - |
| No. of deaths (% of total no. of cases in district) | 3 (13) | 0 (0) | 0 (0) | 3 (38) | 2 (67) | 0 (0) | 2 (50) | - |
| No. of deaths (% of disseminated disease) | 3 (27) | 0 (0) | 0 (0) | 3 (60) | 2 (100) | 0 (0) | 2 (67) | - |
